# Evaluation of Chronotype Among Children and Associations With BMI, Sleep, Anxiety, and Depression

**DOI:** 10.3389/fneur.2020.00416

**Published:** 2020-06-05

**Authors:** Bassam Eid, Mary Bou Saleh, Imad Melki, Paul-Henry Torbey, Joelle Najem, Maroun Saber, Nada El Osta, Lydia Rabbaa Khabbaz

**Affiliations:** ^1^Faculty of Medicine, Saint-Joseph University of Beirut, Beirut, Lebanon; ^2^Department of Pediatrics, Hôtel-Dieu de France Hospital, Saint-Joseph University of Beirut, Beirut, Lebanon; ^3^Laboratoire de Pharmacologie, Pharmacie clinique et Contrôle de qualite des medicaments, Faculty of Pharmacy, Saint-Joseph University of Beirut, Beirut, Lebanon; ^4^Craniofacial Research Laboratory, Oral Health Unit, Faculty of Dental Medicine, Saint-Joseph University, Beirut, Lebanon; ^5^Department of Prosthodontics, Faculty of Dental Medicine, Saint-Joseph University, Beirut, Lebanon

**Keywords:** chronotype, weight, sleep, anxiety, depression, children

## Abstract

**Objectives:** To evaluate possible associations between chronotype, weight, sleep problems, anxiety, and depression among children from 6 to 12 years of age.

**Method:** One-hundred children aged between 6 and 12 years were randomly recruited in five pediatrician clinics in the capital city of Beirut, Lebanon. The protocol was approved by the ethics committee of Saint-Joseph University and Hotel-Dieu Hospital and an informed written formal consent was obtained from one of the parents. The Sleep Disturbance Scale for Children (CCTQ), the Revised Child Anxiety and Depression Scale (RCADS)-Parent version, and the Children's Chronotype Questionnaire (CCTQ) were used.

**Results:** The majority of the sample (47%) presented an intermediate chronotype. There was a shift toward evening chronotype with increased age and a significant association between electronic devices use and an evening chronotype. Higher sleep disturbances were also observed among children with an evening chronotype. In particular, disorders of initiating and maintaining sleep, non-restorative sleep, excessive somnolence, and total SDSC were significantly higher among evening type children in our study. Finally, major depression domain scores were significantly higher among children with an evening chronotype.

**Conclusions:** Several findings of this study are important and explain factors associated to chronotype in children. Two important future perspectives can be highlighted: limiting electronic devices use among children in an effort to reduce circadian rhythm disturbances and identifying and treating sleep problems associated with eveningness, taking into account the possible presence of major depression among this population.

## Introduction

Humans show cyclic rhythmicity in a wide range of psychological, cognitive, and physiological behaviors as well as in hormonal variations. This natural rhythmicity is called the circadian rhythm and it affects several processes such as sleep-wake cycles, mood, hormone levels, cognition, and temperature. The trait determining individual circadian preference in rhythm is known as chronotype, which is relative to cycles of external light and dark (1–5). Chronotypes are divided into three main categories: morning, intermediate, and evening.

Young children (2–6 years of age) seem to show a relatively strong preference for morningness (6–8) but transition toward eveningness begins in early childhood (9) and this shift is significantly more marked in adolescence (10) when a delay in the timing of sleep tends to be seen (11). At the end of the adolescence, a change toward morningness occurs (12). Studies among adolescents and adults have shown that eveningness was significantly associated with obesity when it is compared to morningness (13, 14) and evening chronotype was associated with changes in eating behavior (14–16) such as poor dietary control, high total calories and cholesterol intakes, consumption of a larger portion, late-night food intake, and a tendency to omit breakfast among adults (17, 18). Studies of the links between chronotype and weight among children are very scarce. Only one study was conducted among children with ADHD and revealed that evening preference plays a role in a mechanism linking ADHD to obesity (19). In addition to its possible association with weight, circadian preference was strongly linked with sleep quality in adults (20, 21) and eveningness was related to more sleeping difficulties and in particular to insomnia (22). In pre-school children (aged 4.5 years old), those with an evening chronotype seem to present more sleep difficulties than morning types as reported by parents, and consequently, more negative social consequences (23). Sleep problems were also found to be concurrent with anxiety, depression, conduct problems, and hyperactivity in both children (24, 25) and adolescents (26, 27).

Existing evidence suggests that sleep dysfunction during childhood could be an early manifestation of future adjustment problems (28, 29). Thus, sleep may be used as an early marker of psychopathology developing later and it may enable specialists to identify individuals at risk before the development of more serious symptoms (30). Indeed, among preschool children, sleep problems predicted depression and anxiety at 9–13 years (29). Furthermore, eveningness and sleep difficulties during childhood have been linked to worse academic performance later on, both at school and in university (31) and to adjustment difficulties, defined as internalizing and externalizing problems (32).

Even though several studies examined sleep among children, few of them focused on chronotype, and those were mainly conducted among adolescents. Eveningness has been previously linked to anxiety, depression, and general affective problems among adolescents (33–35) but no such data are available for children.

The period just before adolescence can be critical in determining future sleep pattern and psychological problems and very little is known on the associations between sleep, circadian preference, anxiety, and depression in children during this period. Thus, the primary objective of this study was to investigate the links between the chronotype, weight, sleep problems, anxiety, and depression among children aged between 6 and 12 years.

## Materials and Methods

### Study Design and Population

The study was conducted between March and December 2018. First, five pediatrician clinics were randomly selected from a list of clinics based in Beirut. and randomly recruited in five pediatrician clinics in the capital city of Beirut, Lebanon. Inclusion was done during vaccination visit (before the administration of the vaccine) to make sure that the child is not sick at the time of the inclusion. Briefly, a list of appointments was provided on Monday morning (and for the coming week) by the staff, highlighting vaccination visits. From this list, selection was randomly performed. The exclusion criteria were: children taking any medication, children with any chronic diseases. A trained research assistant interviewed the parents in the waiting room. One-hundred and fifty parent's children were approached, of whom 28 refused to participate in the study, and 22 children presented at least one exclusion criteria. The final study sample consisted of 100 children aged between 6 and 12 years. In addition to filling the questionnaires listed below, socio-demographic information was recorded. Physical activity and electronic devices use were also collected. For physical activity, since the number of participants with no activity (*n* = 3) was small, the two categories of physical activity (no activity and 1 h/week) were combined. The categories were 0–1 h/week, 2 h/week, and ≥3 h/week. For electronic devices use, four categories were used: 0, 1, 2, and ≥3 h/week.

### Sleep Disturbances

The Sleep Disturbance Scale for Children (SDSC) consists of 26 questions evaluating sleep problems during the 6 previous months. SDSC uses a five-point scale and a total sleep time of 1 (9–11 h) to 5 (<5 h) and for sleep latency of 1 (<15 min) to 5 (>60 min). The frequency of the symptoms of sleep disorder is measured on a likers scale of five-point: never (1); occasionally, meaning once or twice per month (2); sometimes, meaning once, or twice per week (3); often, meaning 3–5 times per week (4) and always, meaning six or seven times per week (5). Furthermore, in children, SDSC identifies six types of symptoms of sleep disorders: early and late sleep disorders (sleep latency, sleep duration, nocturnal awakenings, and sleep anxiety), sleep breathing disorders (snoring, breathing) disturbances of arousal (such as sleepwalking, nightmares, and night terrors), disorders of sleep-wake transition (rhythmic movements, hypnotic saccades, and bruxism), disorders of excessive sleepiness (difficulty to wake up, tiredness in the morning, and inappropriate nap) and sleep hyperhidrosis (or night sweating). Calculation of a total score and a score for each symptom is performed for each child. A higher score indicates higher risk of developing sleep disorders (36, 37). The Chronbach alpha of the questionnaire was 0.661.

### Chronotype

The Children's Chronotype Questionnaire (CCTQ) (38) was developed based upon the previous work of Roenneberg et al. (39–41) and Carskadon et al. (42, 43).

The CCTQ is a 27-item, mixed format. Parents can respond to several open-ended questions concerning sleep/wake parameters for scheduled days (non-holidays) and holidays (free days). It has 27 items and a five-point chronotype (CT) score (44). It contains: 16 questions about sleep and wake parameters (e.g., lights-off time, bedtime, sleep latency, rising time, wake-up time, fully alert time, and regular naps) for scheduled and free days; In addition, there is a 10-item morningness/eveningness scale (range of 10–48); and one item number 27 named “chronotype (CT)” (range of 1–5). This is a single-item measure. Parents read a short description of different chronotypes and selected one of five categories that best represents their child's circadian phase preference (i.e., definitely a morning type, rather a morning type than an evening type, neither/nor type, rather an evening type than a morning type, or definitely an evening type).

The total score from the MES [morningness/eveningness (M/E) scale] is a sum of scores of items 17–26 only, and ranges from 10 to 48 (38). This score is used to classify individuals as: morning type, intermediate type, and evening type (scores of ≤ 23, 24–32, and ≥33, respectively) (2). The questionnaire yielded a Cronbach alpha of 0.619.

### RCADS-P

The Revised Child Anxiety and Depression Scale (RCADS)-Parent version is a self-assessment scale developed to identify and screen for the clinical symptoms of anxiety and depression in children or adolescents and is filled by the parents. This questionnaire includes 47 questions grouped into six subscales: Separation Anxiety Disorder, Anxiety Disorder (General Anxiety Disorder), Social Phobia, Panic Disorder, Major Depressive Disorder, and Obsessive Compulsive Disorder. RCADS provides a score for each subscale as well as a total score for anxiety, which is the sum of all anxiety subscales except MDD, a total score for all the scales (sum of six subscales). The higher the score, the more the child presents the clinical symptomatology of anxiety and depression (45, 46). The questionnaire had a Cronbach alpha of 0.759.

### Ethical Considerations

The study protocol obtained the approval of Saint-Joseph University ethics committee of and Hotel-Dieu Hospital ethic committee (USJ, HDF, number CEHDF 1102). Prior to participating, an informed written formal consent was given by one of the parents. The study was conducted between March and December 2018. The self-administered anonymous questionnaires were filled by the children's parent(s).

### Statistical Analysis

The statistical analyses were performed with SPSS software for Windows (version 24.0, Chicago, IL, USA). The level of significance was set at 0.05. The mean and standard deviation were calculated for continuous variables and percentage was calculated for categorical variables. The normality of the distribution of continuous variables was assessed by Kolmogorov-Smirnov tests. In the first step, univariate analyses using the Student's *t*-test or non-parametric Mann-Whitney test and ANOVA (analysis of variance) or its equivalent non-parametric Kruskal-Wallis test were performed. ANOVA (analysis of variance) followed by Tukey *post-hoc* tests or its equivalent non-parametric Kruskal-Wallis test were performed to evaluate the association between continuous variables.

To evaluate the association between continuous variables, Pearson and Spearman correlation coefficients were calculated, and to assess the relationship between categorical variables, Fisher Exact tests, and Chi-square independence tests were performed. In the second step, multiple regression analyses were performed according to the Enter Method; all independent variables with a -*p*- <0.200 were entered into the equation simultaneously. Collinearity among independent variables was also examined. Finally, two regression models were executed; the first model included age, electronic device, social phobia, major depression, separation anxiety, and SDSC. Since, the variables disorders of initiating and maintaining sleep, non-restorative sleep, and sleep breathing disorders are domains that belong to SDSC, they were not included in the same multivariate model. Hence, the second model includes: age, electronic device, social phobia, major depression, separation anxiety, and disorders of maintaining sleep, non-restorative sleep and sleep breathing disorders.

The sample size was calculated according to the formula of Tabachnick and Fidell (47) taking into consideration the number of independent variables to include in the model: the formula used was *N* = 50 + 8*m* (*m* being the number of independent variables for the primary outcome); Given that *m* = 6, we had to include at least 98 subjects in the study.

## Results

### Sociodemographic Characteristics of the Participants

The total number of children included in this study was 100 children. The mean age of the population is 9.10 (±2.25) years old. Twenty percent of the children were overweight and 19% were obese (Table 1).

**Table 1:** Socio-demographic characteristics, eating patterns, physical activity, and electronic devices use of the participants and results obtained for the questionnaires of SDSC, CCTQ, and RCADS-P.

	N^*^	**Minimum**	**Maximum**	**Average**	SD^**^
Age	100	6	12	9.10	2.250
Gender					
Males	38				
Females	62				
BMI (Kg/m^2^)	100	11.0	30.9	19.004	4.118
Underweight	4				
Normal	57				
Obese	19				
Overweight	20				
Number of siblings	100	0	7	1.73	1.145
Crowding index	100	0.4	1.8	1.085	0.3153
At least one of the parents smoke			Premature birth		
No	62		No	94	
Yes	38		Yes	6	
Electronic devices use			Physical activity		
No	45				
1 h/day	20		0–1 h/week……35		
2 h/day	12		2 h/week……61		
≥3 h/day	23		≥3 h/week……4		
Total CCTQ score	100	18	43	29.57	5.487
Chronotype categories					
Evening type	37				
Intermediate	47				
Morning type	16				
Total SDSC score	100	26	60	41.27	8.875
Parasomnias	100	2	23	11.73	4.417
Disorders of initiating and maintaining sleep	100	6	19	10.91	3.232
Sleep breathing disorders	100	3	7	3.66	1.335
Disorders of excessive somnolence	100	2	10	3.11	1.729
Sleep hyperhydrosis	100	3	11	5.85	2.418
Non-restorative sleep	100	4	16	6.01	2.699
Total RCADS-P score	100	12	102	40.32	15.592
Social phobia	100	1	26	12.45	5.709
Panic disorder	100	0	19	3.52	3.789
Separation anxiety	100	0	11	4.34	2.811
Generalized anxiety	100	0	17	7.91	3.861
Obsessive-compulsive disorder	100	0	39	6.51	5.743
Major depression	100	0	14	5.59	3.444
Total anxiety score	100	8	88	34.73	14.424

* *N = % (total N = 100)*;

** *SD, standard deviation*.

Physical activity and electronic devices use are also reported.

### Scores of the CCTQ, SDSC, and RCADS-P Questionnaires

Approximately, half of the sample (47%) were classified as having an intermediate chronotype. The total SDSC and CCTQ scores were respectively 41.27 ± 8.875 and 29.57 ± 5.487. Detailed results are presented in Table 1.

### Association Between Chronotype, BMI, Sleep, and RCADS-P Domains: Results of the Univariate Analysis

Evening type children were significantly older, but no associations were observed between chronotype and BMI (Table 2). Furthermore, there was a significant association between chronotype and electronic devices use: among evening type children, 52% used electronic devices ≥ 3 h/week vs. 4.3% only among morning type children (Table 2). Table 3 shows significant associations between chronotype categories and SDSC or RCADS-P domains and Table 4 presents correlations between chronotype scores and SDSC or RCADS-P domains.

**Table 2:** Association between chronotype categories and participants' characteristics.

		**Chronotype**			*p*-value^***^
		Evening type (N^*^ = 37)	**Intermediate type (*N* = 47)**	**Morning type (*N* = 16)**	
Age (years)	Mean ± SD^**^	9.97 ± 2.279^b^	8.79 ± 2.176^a,b^	8.00 ± 1.713^a^	**0.005**
BMI (Kg/m^2^)	Mean ± SD	19.77 ± 3.976	18.55 ± 4.313	18.55 ± 3.828	0.632
Number of siblings	Mean ± SD	1.65 ± 0.789	1.74 ± 1.343	1.88 ± 1.258	0.801
Gender	Males	17 (44.7%)	18 (47.4%)	3 (7.9%)	0.173
	Females	20 (32.3%)	29 (46.8%)	13 (21.0%)	
Premature birth	No	34 (36.2%)	45 (47.9%)	15 (16.0%)	0.761
	Yes	3 (50.0%)	2 (33.3%)	1 (16.7%)	
Electronic devices use	No	9 (20.0%)	25 (55.6%)	11 (24.4%)	**0.009**
	1 h/week	7 (35.0%)	10 (50.0%)	3 (15.0%)	
	2 h/week	9 (75.0%)	2 (16.7%)	1 (8.3%)	
	≥3 h/week	12 (52.2%)	10 (43.5%)	1 (4.3%)	
Physical activity	≤ 1 h/week	15 (40.5%)	18 (38.3%)	2 (12.5%)	**0.217**
	2 h/week	22 (36.1%)	27 (44.3%)	12 (19.7%)	
	3–4 h/week	0 (0%)	2 (50.0%)	2 (50.0%)	
At least one parent smokes	No	22 (35.5%)	27 (43.5%)	13 (21.0%)	0.220
	Yes	15 (39.5%)	20 (52.6%)	3 (7.9%)	

* *N = % (total N = 100)*;

** *SD, standard deviation*;

*** *p-values in bold are significant*.

*Chi-Square tests and Fisher Exact tests for the comparisons of categorical variables*.

*ANOVA followed by Tukey post-hoc tests and Kruskal-Wallis tests for the comparison of continuous variables*.

a,b *Different letters indicate the presence of a significant difference according to Tukey post-hoc tests. Bold values are significant*.

**Table 3:** Associations between chronotype categories, SDSC, and RCADS-P domains (*N* = 100).

	**CCTQ**	N^*^	**Average**	SD^**^	***p*-value**
Disorders of initiating and maintaining sleep	Evening type	37	11.46^b^	2.950	0.005
	Intermediate type	47	11.28^b^	3.450	
	Morning type	16	8.56^a^	2.128	
Disorders of excessive somnolence	Evening type	37	3.70^b^	2.259	0.020
	Intermediate type	47	2.87^a^	1.296	
	Morning type	16	2.44^a^	0.892	
Non-restorative sleep	Evening type	37	6.97^b^	3.655	0.019
	Intermediate type	47	5.55^a^	1.767	
	Morning type	16	5.12^a^	1.628	
Total SDSC score	Evening type	37	44.14^b^	8.331	<0.000
	Intermediate type	47	41.47^b^	8.637	
	Morning type	16	34.06^a^	6.981	
Social phobia	Evening type	37	10.35^a^	4.996	0.005
	Intermediate type	47	14.32^b^	5.809	
	Morning type	16	11.81^a,b^	5.431	
Major depression	Evening type	37	6.89^b^	3.332	0.002
	Intermediate type	47	5.28^a,b^	3.437	
	Morning type	16	3.50^a^	2.503	
Separation anxiety	Evening type	37	3.24^a^	2.510	0.009
	Intermediate type	47	4.89^a,b^	2.846	
	Morning type	16	5.25^b^	2.720	

*Only significant associations are shown in the table (-p-value < 0.05)*.

* *N = % (total N = 100)*;

** *SD, standard deviation*.

*ANOVA followed by Tukey post-hoc tests and Kruskal-Wallis tests for the comparison of continuous variables*.

a,b *Different letters indicate the presence of a significant difference according to Tukey post-hoc tests*.

**Table 4:** Correlations between SDSC, CCTQ, and RCADS-P scores (*N* = 100).

		**TAS**	**TS**	**SP**	**PD**	**MD**	**SA**	**GA**	**OCD**	**PS**	**DIMS**	**SBD**	**DES**	**SH**	**NRS**	**SDSC**
Total anxiety score (TAS)	CC	1														
	-*p*-value															
Total RCADS-P score (TS)	CC	**0.977**	1													
	-*p*-value	**<0.000**														
SP	CC	**0.559**	**0.496**	1												
	-*p*-value	**<0.000**	**<0.000**													
PD	CC	**0.711**	**0.747**	0.068	1											
	-*p*-value	**<0.000**	**<0.000**	0.501												
MD	CC	**0.234**	**0.437**	−0.095	**0.407**	1										
	-*p*-value	**0.019**	**<0.000**	0.345	**<0.000**											
SA	CC	**0.476**	**0.431**	**0.321**	0.102	−0.039	1									
	-*p*-value	**<0.000**	**<0.000**	**0.001**	0.314	0.703										
GA	CC	**0.684**	**0.674**	0.169	**0.476**	0.187	0.128	1								
	-*p*-value	**<0.000**	**<0.000**	0.094	**<0.000**	0.062	0.203									
OCD	CC	**0.795**	**0.803**	0.093	**0.688**	**0.306**	**0.232**	**0.501**	1							
	-*p*-value	**<0.000**	**<0.000**	0.357	**<0.000**	**0.002**	**0.020**	**<0.000**								
Parasomnias (PS)	CC	**0.332**	**0.393**	0.139	**0.258**	**0.390**	0.026	0.202	**0.377**	1						
	-*p*-value	**0.001**	**<0.000**	0.169	**0.010**	**<0.000**	0.796	0.044	**<0.000**							
Disorder initiating maintaining sleep (DIMS)	CC	−0.025	0.029	0.056	−0.108	**0.235**	0.175	−0.039	−0.107	0.151	1					
	-*p*-value	0.803	0.777	0.581	0.283	**018**	0.082	0.702	0.290	0.133						
Sleep breathing disorders (SBD)	CC	**0.335**	**0.363**	0.039	**0.261**	**0.242**	−0.042	**0.349**	**0.415**	0.137	−0.138	1				
	-*p*-value	**0.001**	**<0.000**	0.701	**0.009**	**0.015**	0.681	**<0.000**	**<0.000**	0.175	0.170					
Disorder excessive somnolence (DES)	CC	−0.180	−0.141	–**0.206**	−0.152	0.113	−0.047	−0.162	−0.015	**0.321**	0.179	**0.199**	1			
	-*p*-value	0.073	0.160	**0.040**	0.130	0.264	0.640	0.107	0.883	**0.001**	0.075	**0.047**				
Sleep hyperhydrosis (SH)	CC	0.154	**0.207**	**0.141**	0.046	**0.221**	0.014	0.124	0.126	**0.434**	−0.002	**0.325**	**0.305**	1		
	-*p*-value	0.126	**0.039**	0.162	0.649	**0.027**	0.894	0.219	0.210	**<0.000**	0.986	**0.001**	**0.002**			
Non-restorative sleep (NRS)	CC	–**0.286**	–**0.240**	–**0.262**	−0.128	0.113	−0.190	–**0.246**	−0.116	**0.234**	0.048	0.040	**0.422**	**0.264**	1	
	-*p*-value	**0.004**	**0.016**	**0.008**	0.205	0.261	0.059	**0.014**	0.249	**0.019**	0.638	0.691	**<0.000**	**0.008**		
Total SDSC	CC	0.126	**0.212**	0.014	0.072	**0.433**	0.007	0.066	**0.208**	**0.746**	**0.467**	**0.293**	**0.551**	**0.605**	**0.468**	1
	-*p*-value	0.211	**0.034**	0.891	0.476	**<0.000**	0.943	0.513	**0.038**	**<0.000**	**<0.000**	**0.003**	**<0.000**	**<0.000**	**<0.000**	
**CCTQ score**	CC	−0.114	−0.030	–**0.201**	0.035	**0.343**	–**0.248**	0.105	−0.059	0.067	**0.326**	**0.200**	0.186	0.035	**0.241**	**0.301**
	-*p*-value	0.257	0.766	**0.044**	0.732	**<0.000**	**0.013**	0.299	0.558	0.509	**0.001**	**0.046**	0.064	0.726	**0.016**	**0.002**

*Pearson and Spearman correlation coefficients were calculated to assess the relationship between categorical variables. The CC is used for Spearman and Pearson Correlation Coefficient. SDSC, Sleep Disturbance Scale for Children; RCADS-P, Revised Child Anxiety and Depression Scale parent version; SP, Social phobia; PD, panic disorder; SA, separation anxiety; GD, generalized anxiety; OCD, obsessive-compulsive disorder; MD, major depression; TAS, total anxiety score; CC, correlation coefficient. Values in bold are significant. Pearson and Spearman correlation coefficients*.

### Association Between Chronotype, BMI, Sleep, and RCADS-P Domains: Results of the Multivariate Analysis

Later chronotype (higher CCTQ score) was associated with higher SDSC score, indicating greater sleep disturbance; It was also associated with older age, electronic devices use and with higher score on the major depression domain of the RCADS-P (Table 5).

**Table 5:** Multivariate analysis according to Enter Method: chronotype score taken as the dependent variable.

	**Unstandardized coefficients**		**Standardized coefficients**	***t***	**Sig**.	**95.0% Confidence Interval for** ***B***	
	***B***	**Standard error**	**Beta**			**Lower bound**	**Upper bound**
Age	0.497	0.236	0.204	2.104	0.038	0.028	0.966
Electronic devices use	1.034	0.473	0.230	2.185	0.031	0.095	1.973
Social phobia	−0.107	0.092	−0.112	−1.160	0.249	−0.291	0.076
Major depression	0.449	0.149	0.282	3.017	0.003	0.154	0.744
Separation anxiety	−0.281	0.194	−0.144	−1.446	0.151	−0.667	0.105
SDSC total score	0.169	0.055	0.273	3.052	0.003	0.059	0.279
Age	0.513	0.239	0.210	2.141	0.035	0.037	0.988
Electronic devices use	1.016	0.463	0.226	2.192	0.031	0.095	1.936
Social phobia	−0.111	0.088	−0.116	−1.269	0.208	−0.286	0.063
Major depression	0.207	0.149	0.130	1.384	0.170	−0.090	0.503
Separation anxiety	−0.330	0.187	−0.169	−1.766	0.081	−0.702	0.041
Disorders of initiating and maintaining sleep	0.530	0.156	0.312	3.406	0.001	0.221	0.839
Non-restorative sleep	0.226	0.179	0.111	1.265	0.209	−0.129	0.582
Sleep breathing disorders	1.072	0.385	0.261	2.784	0.007	0.307	1.837

*SDSC: Sleep Disturbance Scale for Children*.

## Discussion

Our results showed that the majority of the sample (47%) presented an intermediate chronotype which is consistent with several previously conducted studies (42, 48); However, 37% of the sample presented an evening chronotype, which was higher than other studies (48). This could be explained by the fact that different instruments are used, such as the youth self-report with the Morningness/Eveningness Scale in Children (MESC) in the study of Carskadon et al. (42). Furthermore, differences in age and gender distribution exist between the different study's samples. Among our sample, the average age was around 9 years old, which is closer to puberty onset, especially in females (consisting 62% of our sample). Indeed, it was previously reported that the onset of puberty triggers an evening preference among approximately 40% of youth, which is compounded by social changes (e.g., less parental control, technology) (49, 50). In addition, the chronotype depends on genetic and environmental factors (51) and these factors are specific to each population and each culture.

There was a shift toward evening chronotype with increased age: evening type children were significantly older than morning type (9.97 ± 2.279 vs. 8.00 ± 1.713 years of age). It was previously reported that a shift toward later sleep rhythm occurs from early to late adolescence (48, 52) and eveningness was associated with older participants in another recent study conducted among participants aged from 11 to 19 years (48).

Excessive use of electronic media (such as computers, tablets, smartphones, gaming consoles, etc.) among adolescents was known to be associated with a disruption in the circadian clock, irregular, shortened, and later sleep onset (53). Little is known about this topic among children, but one could predict that the same consequences would be seen. Indeed, our results showed a significant association between electronic devices use and an evening chronotype.

Higher sleep disturbances were also observed among children with an evening chronotype, similarily to previous report about eveningness among youth resulting in sleep deprivation (49, 50). Several authors previously noted that an evening preference was associated with an irregular sleep-wake schedule (54). In particular, disorders of initiating and maintaining sleep, non-restorative sleep, excessive somnolence, and total SDSC were significantly higher among evening type children in our study. In fact, the most common symptom of sleep disorders is non-restorative sleep, which results in daytime sleepiness (55). Several studies demonstrated that non-restorative sleep is associated with other various health problems such as heart disease, respiratory diseases, obesity, depressive symptoms, and suicide among adults (56). Non-restorative sleep and a short sleep duration were significantly linked to suicidal ideation in adolescents (57). Very few data exist about the consequences of non-restorative sleep among children but they are probably problematic. Thus, interventions aiming at screening sleep problems, especially non-restorative sleep associated with eveningness among children seem important because they might prevent further sleep complications later on in life.

Finally, anxiety domains of the RCADS-P were not associated to chronotype after performing the multivariate analysis but major depression scores were significantly higher among children with an evening chronotype (38–44). Exhibiting preference for eveningness has already been associated with several negative outcomes, in particular, depression (58–64), poor academic performance (34, 61), physical inactivity, higher rates of alcohol use, of smoking, and obesity (65, 66) among adolescents and adults. A recent report among children and adolescents between 11 and 19 years old (42) also showed that eveningness was associated with higher levels of depression.

The reciprocal effects between chronotype and depression are poorly understood during the critical developmental period extending from childhood to adolescence and the nature of our study (cross-sectional) does not allow to draw hypothesis on the cause-effect relationship between chronotype and depression. In addition, the nature of the questionnaire used (RCADS-P), which is a screening and not a diagnostic tool, does not allow to assume a diagnosis of major depression, only a high suspicion of it.

Our findings should take into account several limitations. The results were obtained through questionnaires filled out by the parent of the child. Although, parents are aware of when they put their children to bed and when they wake up, the ability of parents to report on their child's preference for evening vs. morning is more limited. No information about Tanner Stage was available even though it could affect chronotype. The cross-sectional design of this study limits the authors' ability to determine whether later chronotype preceded or followed the existence of high depression scores on the RCADS-P; thus, the methodology is not adapted to establish a causal relationship between chronotype and depression suspicion. Finally, we excluded any chronic disease and of chronic medication intake, which prevented us from studying the impact of comorbidities on chronotype.

Despite these limitations, several findings observed in this study explain factors associated to circadian rhythm disturbances in children; the aspects explored are important and warrant further investigations.

## Conclusion

Significant associations were observed in this study between chronotype and sleep as well as between chronotype and major depression domain of the RCADS-P among children. Based on our results, two important future perspectives can be highlighted: (i) limiting electronic devices use among children in an effort to reduce circadian rhythm disturbances and (ii) identifying and treating sleep problems associated with eveningness, taking into account the possible presence of major depression among this population.

## Data Availability Statement

The datasets generated for this study are available on request to the corresponding author.

## Ethics Statement

The studies involving human participants were reviewed and approved by ethics committee of Saint-Joseph University and Hotel-Dieu Hospital. Written informed consent to participate in this study was provided by the participants' legal guardian/next of kin.

## Author Contributions

BE and MB: data collection and analysis. P-HT and IM: data collection and protocol design. NE: statistical analysis and protocol design. JN and MS: data collection and writing manuscript draft. LR: protocol design, interpretation of data, writing the draft, and final manuscript.

## Conflict of Interest

The authors declare that the research was conducted in the absence of any commercial or financial relationships that could be construed as a potential conflict of interest.
